# Dynamic modulation of the non-canonical NF-κB signaling pathway for HIV shock and kill

**DOI:** 10.3389/fcimb.2024.1354502

**Published:** 2024-03-05

**Authors:** Aswath P. Chandrasekar, Mark Maynes, Andrew D. Badley

**Affiliations:** ^1^ Department of Laboratory Medicine and Pathology, Mayo Clinic, Rochester MN, United States; ^2^ Division of Infectious Diseases, Mayo Clinic, Rochester, MN, United States; ^3^ Department of Immunology, Mayo Clinic, Rochester, MN, United States; ^4^ Graduate School of Biomedical Sciences, Mayo Clinic, Rochester, MN, United States; ^5^ Department of Molecular Medicine, Mayo Clinic, Rochester, MN, United States

**Keywords:** HIV, NFkappa B signaling pathway, shock and kill strategies, HIV cure strategies, latency reversal agents

## Abstract

HIV cure still remains an elusive target. The “Shock and Kill” strategy which aims to reactivate HIV from latently infected cells and subsequently kill them through virally induced apoptosis or immune mediated clearance, is the subject of widespread investigation. NF-κB is a ubiquitous transcription factor which serves as a point of confluence for a number of intracellular signaling pathways and is also a crucial regulator of HIV transcription. Due to its relatively lower side effect profile and proven role in HIV transcription, the non-canonical NF-κB pathway has emerged as an attractive target for HIV reactivation, as a first step towards eradication. A comprehensive review examining this pathway in the setting of HIV and its potential utility to cure efforts is currently lacking. This review aims to summarize non-canonical NF-κB signaling and the importance of this pathway in HIV shock-and-kill efforts.

## Introduction

The HIV latent reservoir refers to the pool of integrated-but-transcriptionally-silent HIV pro-viral DNA that persists indefinitely *in vivo* (Siliciano and Greene, 2011). The latent reservoir begins to seed within days of acute HIV infection. Combination anti-retroviral therapy (ART) can suppress viral replication and plasma viremia to undetectable levels; however, treatment interruption and ensuing reservoir reactivation allow for viral rebound and the return of plasma viremia (Vanhamel et al., 2019). The exact mechanisms underlying this partial and selective transcriptional silencing are poorly understood, and it has been observed that multiple factors contribute to determining the size of the reservoir including the efficacy of the host immune response, the level of plasma viremia in acute infection and the interval between infection and anti-HIV therapy initiation. Additionally, it has now become apparent that even under the cover of suppressive ART (which is capable of reducing plasma viremia to undetectable levels), the HIV reservoir remains dynamic with low-level replication and reservoir seeding through clonal expansion (Yeh et al., 2021; Hosmane et al., 2017; Bui et al., 2017; Bachmann et al., 2019; Woldemeskel et al., 2020).

A cure for HIV may be achieved either as a “sterilizing cure”, which is the complete eradication of the virus; or a “functional cure”, achieving the absence of viremia without ART. Within this paradigm, the “shock and kill” theory is currently under investigation at various levels as a feasible sterilizing or functional cure strategy. Briefly, shock and kill therapy aims to therapeutically reactivate latent HIV and subsequently clear it through a combination of immune mechanisms, the cytopathic effects of viral reactivation, and drugs that selectively eradicate reactivating cells (Kim et al., 2018; Chandrasekar and Badley, 2022). Importantly, identifying a feasible shock agent that potently activates HIV remains the major objective. Furthermore, learning the various mechanisms that drive HIV replication is central to the shock and kill strategy. An understanding of key transcriptional regulators is necessary to identify viable drug candidates that may reactivate the latent reservoir. The Nuclear Factor Kappa Light Chain Enhancer of B Cells (NF-κB) signaling pathway has long been recognized as a major driver of HIV replication and its relationship to HIV transcription has been extensively studied (Hiscott et al., 2001). Between the canonical and non-canonical pathways that the NF-κB family can signal through, studies are now beginning to identify the non-canonical NF-κB pathway as a potential target for HIV cure efforts. This review aims to examine the non-canonical NF-κB pathway in depth and provide a comprehensive resource that would help inform future investigations.

## The NF-κB Family

The Nuclear Factor Kappa Light Chain Enhancer of B Cells (NF-κB) complex constitutes one of the key transcription regulatory factor families, and is present in almost all cells, across most living higher organisms (Gilmore, 2006). The NF-κB family, defined by the presence of a conserved homology domain known as “Rel”, includes five individual Rel containing proteins – NF-KB1, NF-KB2, RelA, RelB and c-Rel (O'Dea and Hoffmann, 2010). These proteins interact with each other to form dimers that are capable of binding to DNA and activating transcription.

The NF-κB1 and NF-κB2 proteins in their inactive states are termed p105 and p100, respectively. Following activation or as a result of translational arrest, these proteins are transformed to their active, shorter conformations – p50 and p52 through protease mediated cleavage. The p50 and p52 proteins can form dimers either with each other, with themselves or with the RelA (p65), RelB and c-Rel members of the family and exert downstream effects (Gilmore, 2006; O'Dea and Hoffmann, 2010; Hoffmann and Baltimore, 2006). Of the possible dimer combinations, the combinations of p50:p50, p52:p52 and p50:p52, while capable of binding to DNA, are not known to exert transcriptional regulation. Additionally, the RelA : RelB, c-Rel : RelB and RelB : RelB dimers are incapable of binding to DNA. The remaining possible combinations are all capable of initiating transcription (O'Dea and Hoffmann, 2010).

NF-κB1 and 2 are, at baseline, in an inactive state; maintained as such, by their association with a group of regulatory, inhibitory proteins known as Inhibitory Kappa B (IκB). This family can further be subclassified into the typical IκB proteins: IκBα, IκBβ and IκBϵ, and the atypical IκB proteins: BCL-3, IκBξ and IκBNS (Tam and Sen, 2001; Whiteside et al., 1997; Nolan et al., 1993; Yu et al., 2020). These proteins bind to the NF-κB precursor proteins p105 and p100 and prevent their cleavage into their active forms. The Inhibitory Kappa B Kinase complex (IKK), consisting of the enzymes IKKα, IKKβ and IKKγ (also called NF-κB essential modifier (NEMO)), bind, phosphorylate and cause the degradation of the IκB proteins, allowing for p52 or p50 release and downstream signaling (Scheidereit, 2006).

Through exogenous or endogenous initiators, the NF-κB signaling cascade may be activated, leading to the regulation of cellular transcription and the up or down regulation of protein production. There may be numerous cascades through which NF-κB signaling may occur, but broadly, two major signaling pathways exist: a canonical NF-κB (cNF-κB) pathway and non-canonical NF-κB (ncNF-κB) pathway (Gilmore, 2006; O'Dea and Hoffmann, 2010; Hoffmann and Baltimore, 2006; Pomerantz and Baltimore, 2002). The cNF-κB pathway involves NF-κB1 protein in a p50:RelA (p65) heterodimer that is capable of binding to DNA and inducing transcriptional regulation and is largely the most constitutively active NF-κB signaling cascade. A comprehensive review of the mechanics and effects of the cNF-κB cascade is beyond the scope of this article, [reviewed in detail in (Yu et al., 2020)], but briefly, canonical NF-κB signaling may be initiated through multiple mechanisms including T cell receptors, B-cell receptors, cytokine receptors, and innate pattern recognition receptors. Canonical signaling promotes immune cell activation, the production of pro-inflammatory cytokines, angiogenesis and leads to immune recruitment. Dysregulated cNF-κB signaling has been described as a pathogenic mechanism in autoimmune diseases and malignancy (Yu et al., 2020). The ncNF-κB pathway involves the NF-κB2 protein in a p52:RelB heterodimer that is examined in detail below.

## The non-canonical NF-κB pathway and its regulation

The Non-canonical NF-κB pathway culminates in transcriptional regulation by the NF-κB2 protein, in a p52:RelB heterodimer. At baseline, the p100 protein restricts RelB activity, functioning like a IκB protein. Following proteolysis to p52 and dimerization with RelB, the complex undergoes nuclear translocation and regulates transcription (Sun, 2011). The central component to ncNF-κB signaling is the NF-κB inducing kinase (NIK), a MAP3K like protein, which is a potent and specific inducer of p100 processing. NIK activation leads to downstream phosphorylation, ubiquitination, and activation of the ncNF-κB cascade through IKKα activation, and potentiation of IKKα-p100 binding (Senftleben et al., 2001; Xiao et al., 2004; Xiao et al., 2001). At cellular homeostasis, p100 is conjugated to the SUMO1 protein in a post-translational SUMOylating process, mediated through the Ubc9 enzyme, a process critical to normal p100 processing (Vatsyayan et al., 2008).

The activation the ncNF-κB cascade may be initiated by receptor-ligand interaction on the cell surface. Specifically, signaling through tumor necrosis factor (TNF) superfamily members and their receptors such as: CD40, B-cell activating factor (BAFFR), Lymphotoxin β Receptor (LTβR), Receptor activator for NF-κB (RANK), TNFR2, Fibroblast growth factor inducible factor (FN14), CD27, CD30, or OX40 (CD134) have been shown to activate the ncNF-κB. Additionally, signaling through the macrophage colony stimulating factor receptor (MCSFR) or membrane attack complexes (MACs) have also been shown to activate the ncNF-κB. Activation of the retinoic acid inducible gene 1 (RIG-1) by viral pathogens has also been shown to activate the ncNF-κB (Sun, 2011; Sun, 2017).

ncNF-κB activation through cell surface receptor-ligand interactions are primarily dependent on the recruitment and degradation of the TNF receptor associated factors (TRAF) 2 and 3. TRAF2 and TRAF3, in conjunction with the cellular inhibitor of apoptosis-1&2 (cIAP1 & cIAP2) proteins, function as the main negative regulators of ncNF-κB signaling (Vallabhapurapu et al., 2008; Zarnegar et al., 2008). In the inactivated state, TRAF3 is bound to NIK leading to its ubiquitination and proteasomal degradation, preventing p52 processing. TRAF3-NIK binding is mediated by the cIAP protein in a TRAF2-dependent manner. Following receptor ligation, activated cIAP mediates TRAF3 degradation, which allows for the release and accumulation of NIK (Sun, 2011; 2017). This process may be positively or negatively regulated *in vivo*.

### Positive regulation of the non-canonical pathway

Positive regulation of the cascade may be mediated by receptor ligand interaction or by intracellular proteins. Increased ligand-receptor interaction would likely lead to increased signaling. Intracellularly, NIK activation may also be positively regulated by cytoplasmic proteins such as Zfp91, which leads to NIK stabilization and downstream p100 processing (Jin et al., 2010). Positive regulation of NIK may also occur through MALT-1, which promotes TRAF3 ubiquitination (Sun, 2011), or BCL-10, which promotes NIK phosphorylation (Bhattacharyya et al., 2010). Additionally, independent activation of IKKα by proteins such as STAT3 have also been shown to induce p100 processing. Another key regulatory step in the ncNF-κB cascade is the NIK induced processing of p100 which is mediated through the binding of βTRcP to p100 (following its phosphorylation at serine residues 866 and 870) and subsequent ubiquitination and degradation (Yu et al., 2020). At the nuclear level, p100 degradation may be mediated by the Fbw7 protein (Fukushima et al., 2012).

### Negative regulation of the non-canonical pathway

The ncNF-κB cascade is negatively regulated at various steps in the pathway. As mentioned above, at baseline, the TRAF3-TRAF2-cIAP complex prevents the intracellular accumulation and activation of NIK; however, even following receptor-ligand binding, the TRAF3 ubiquitination process may be inhibited by the cytoplasmic deubiquitinase belonging to the OUT family, such as A20 and OTUD7B, attenuating the pathway (Hu et al., 2013; Pujari et al., 2013). NIK induced IKKα activation may independently lead to NIK destabilization by IKKα in a negative feedback mechanism (Razani et al., 2010). Intracytoplasmic proteins such as NLRP12 (Allen et al., 2012) and Tank binding kinase (TBK1) may lead to NIK degradation (Jin et al., 2012). In neural tissues, TRIM9 has been shown to inhibit NIK mediated p100 processing (Shi et al., 2014). IKKα may also be independently inhibited by specific microRNAs.

Ultimately, the accumulation of stable NIK leads to IKKα activation and p100 processing. The released p52 protein associates with RelB and translocates to the nucleus where it regulates transcription, binding to specific κB sites. It has been suggested that there exists similar DNA-binding specificity of canonical and non-canonical NF-κB members (Britanova et al., 2008).

**Figure 1:**
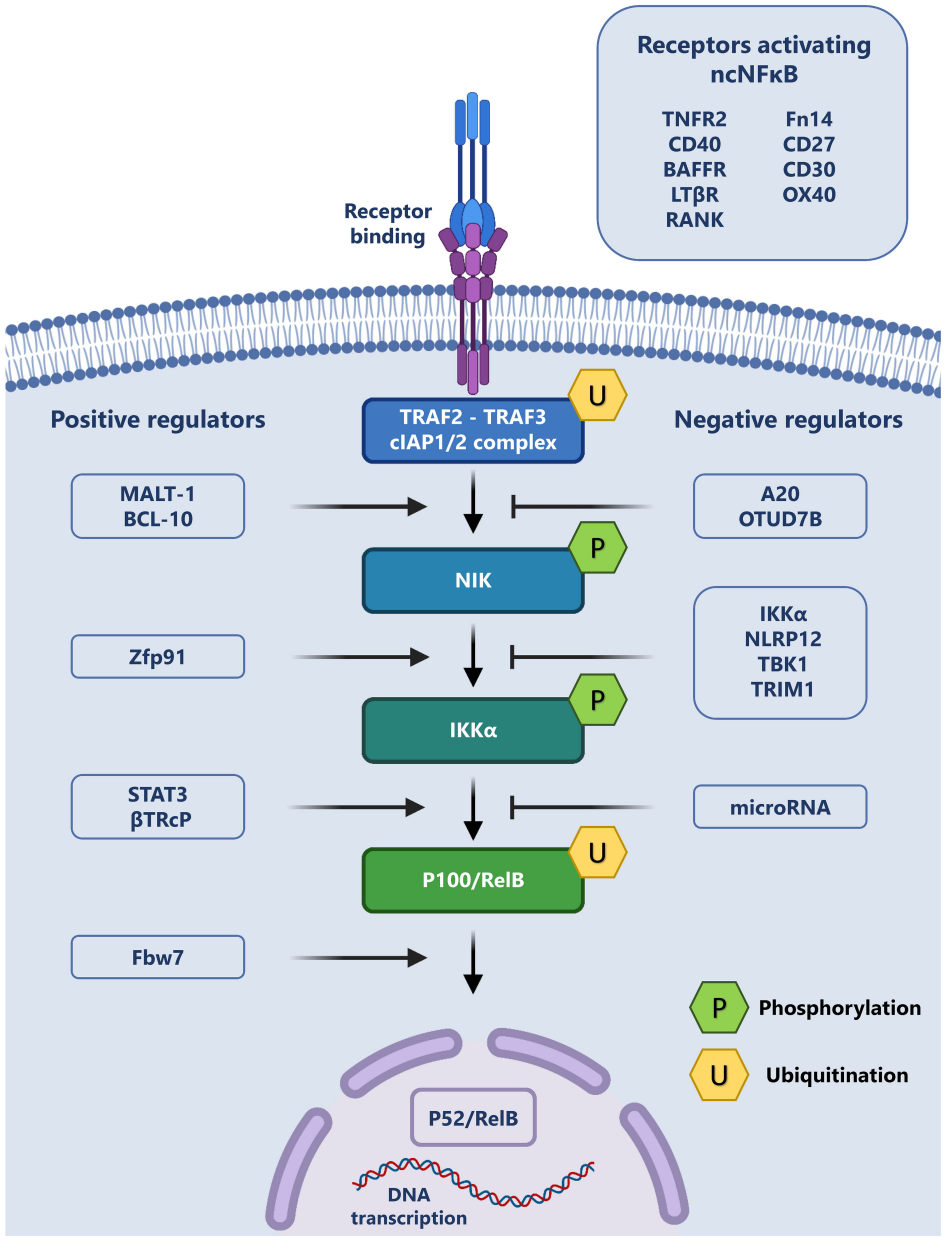
The Non-canonical NF-κB pathway and its regulation: The non-canonical signaling pathway may be initiated through the binding of a variety of exogenous ligands to their cognate receptors (top right). This ligand receptor interaction leads to the recruitment of TRAF2, TRAF3 and cIAP1/2, forming a complex, which allows for the release and phosphorylation of NIK. Phospho-NIK is able to in turn phosphorylate IKKα, which leads to the ubiquitination of the p100/RelB complex and p52 production. P52 subsequently translocates to the nucleus and regulates transcription. This cascade may be positively regulated (left) or negatively regulated (right) by a variety of intracellular proteins.

A summary of the non-canonical pathway and the involved receptors and regulators is provided in **Figure 1**.

## Non-canonical NF-κB regulators and the effects of HIV infection

While at the population level, there is wide variance to sequences into which HIV integrates, some regions have been identified as preferential sites of integration, and having bearing on HIV persistence, (Reviewed in (Hughes and Coffin, 2016)). These include regions with transcription-associated histone modifications and regions that correspond to specific mutations in HIV integrase (Hughes and Coffin, 2016; Wang et al., 2007; Serrao et al., 2014). Serving as a major transcriptional regulator, the role of NF-κB in HIV transcription during active infection has been extensively studied (reviewed in (Hiscott et al., 2001)) and is essential for efficient replication. Additionally, it has been demonstrated that the long terminal repeat (LTR) of the HIV genome harbors two independent NF-κB binding sites that are essential for normal HIV transcription and replication, and cells with lower levels of NF-κB may facilitate the establishment of HIV latency (Hiscott et al., 2001; Jiang and Dandekar, 2015; Kwon et al., 1998; Alcamí et al., 1995). CD4+ T cells (which harbor the vast majority of the latent reservoir) are described to have very low levels of NF-κB activity at baseline, and this is mostly mediated through the non-canonical p50 pathway (Hiscott et al., 2001). It is widely accepted that memory CD4+ T Cells harbor the vast majority of the latent reservoir and therefore, understanding of the infection-induced modifications to the regulators of the ncNF-κB pathway becomes essential to inform cure studies and will be examined here, and are summarized in **Table 1**.

**Table 1:** The known effects of non-canonical NF-κB regulators in HIV infection.

Non-canonical Regulators	Role in non-canonical signaling	Known effects in HIV infection	References
TNFR2	Extracellular receptor	Inhibits HIV entry and thereby replication. Reverses HIV latency	(Pasquereau et al., 2017)
CD40-CD40L	Extracellular receptor	Blockade inhibits latency reversal	(Kornbluth, 2000; Donhauser et al., 2012; Kristoff et al., 2019)
BAFF	Extracellular receptor	Serum levels elevated in active infection, reversible with ART. Elevated levels on Monocyte derived macrophages in HIV infection	(Gomez et al., 2016; Fontaine et al., 2011)
LTβR	Extracellular receptor	Increases HIV replication. HIV Tat induced LTα could induce ncNF-κB	(Sastry et al., 1990; Marshall et al., 1999; Schmidt et al., 2021)
RANK	Extracellular receptor	Drives HIV replication in an NF-κB dependent fashion	(Kelesidis et al., 2014)
CD27	Extracellular receptor	Serum levels elevated in HIV infection	(Widney et al., 1999)
CD30	Extracellular receptor	HIV RNA is enriched in CD30^+^ CD4^+^ cells *in vivo*. CD30 blockade led to significantly reduced HIV DNA	(Biswas et al., 1995; Biswas et al., 2006; Hogan et al., 2018)
OX40	Extracellular receptor	Drives HIV replication in an NF-κB dependent manner. OX40^+^ CD4^+^ cells harbor significantly higher intact HIV virions	(Takahashi et al., 2001; Kuo et al., 2018)
NIK	Key intracellular regulator protein	Favors HIV replication mediated through HIV Tat	(Li et al., 2001; Zhou et al., 2008)
cIAP2	Key intracellular regulator protein	Negatively regulates HIV transcription	(Pache et al., 2015)
STAT3	Positive regulatory protein	HIV GP120: STAT3 mediated NF-κB activation HIV Nef: STAT3 activation and phosphorylation HIV Tat: STAT3 phosphorylation, dimerization, and nuclear translocation	(Briggs et al., 2001; Cicala et al., 2002; Husain et al., 2005; Zeng et al., 2007; Jarboui et al., 2012; Del Cornò et al., 2014; Pache et al., 2015)
Zfp91	Positive regulatory protein	Tat mediated HIV transcription	(Faust et al., 2018)
MALT1	Positive regulatory protein	Pro-replication profile	(Liu et al., 2013; Li H. et al., 2016)
RIG1	Positive regulatory protein	Decreased cytoplasmic RIG1 levels due to HIV protease	(Solis et al., 2011; Zahoor et al., 2014)
TBK1	Negative regulatory protein	HIV Vif and Vpr: prevent TBK1 autophosphorylation and the formation of a TRAF3-TBK1-IRF3 complex, antagonizing interferon production	(Harman et al., 2015)
A20	Negative regulatory protein	Upregulated in intestinal epithelial cells following ART	(Chitre et al., 2018)

In acute and chronic infected states, HIV and its proteins may affect the ncNF-κB pathway through modulation of regulatory proteins, leading to increased or decreased HIV transcription.

### Surface receptor modulation

As mentioned above, non-canonical signaling may be initiated through receptor ligand interactions at the cell surface. Chronic infection may up or downregulate the expression of these receptors, leading to potential pathogenic ramifications downstream.

Tumor Necrosis Factor Receptor 2 (TNFR2) stimulation activates both the canonical and non-canonical NF-κB pathways. TNF-mediated stimulation has been shown to reactivate HIV in *in-vitro* models of latency and in *ex vivo* studies in combination with other latency reversal agents but has been seen to be associated with significant toxicity to bystander cells. TNFR2 signaling may also control HIV replication through inhibition of HIV entry through CD4 downregulation (Pasquereau et al., 2017).

CD40-CD40L binding in active HIV infection contributes to HIV control through the regulation of chemokine secretion, antibody production and immune effector function. The levels of plasma and surface expression of CD40 have been observed to vary in acute, chronic, and treated infection (Kornbluth, 2000; Donhauser et al., 2012). With respect to shock and kill strategies, it has been observed that CD40L-CD40R blockade was sufficient to significantly reduce HIV latency reversal and protein production in a myeloid dendritic cell induced model (Kristoff et al., 2019).

B-cell activating factor (BAFF) is primarily produced on the surface of antigen-presenting cells of myeloid lineage such as monocytes and dendritic cells. HIV has been demonstrated to independently upregulate BAFF expression in monocyte derived macrophages (Gomez et al., 2016). In HIV infection, serum BAFF levels have been seen to increase steadily over the duration of infection, and in animal models, this increase was reversible with ART (Fontaine et al., 2011). The BAFF/BAFFR axis, while primarily involved in B-Cell regulation, has been demonstrated to influence T cell activation and proliferation (Huard et al., 2001; Ye et al., 2004). In HIV infection, therefore, BAFF upregulation on the surface of dendritic cells and B-Cells as well as secreted BAFF in the microenvironment could exert pro-transcriptional activity and drive HIV replication.

Lymphotoxin β Receptor (LTβR) signaling is known to increase HIV replication alone and in the presence of TNFα (Marshall et al., 1999). It has been established that Naïve T cells are the major producers of LTβ in lymphoid tissues and that HIV-associated CD4+ T cell depletion leads to dysregulation of normal immune architecture, concurrent with depleted LTβR signaling, an effect that was seen to be reversible with ART (Zeng et al., 2012). HIV Tat has also been shown to induce the production of Lymphotoxin α (LTα), which may also signal through the LTβR and drive non-canonical NF-κB activation. However, considering the observed off-target effect of therapeutic targeting of this receptor, further research is necessary to improve its feasibility as a safe target for latency-reversal therapy (Sastry et al., 1990; Schmidt et al., 2021).

Receptor activator for NF-κB (RANK), RANK ligand (RANKL) and its soluble receptor Osteoprotegerin (OPG) may be up or down regulated during active HIV infection or following ART which has been extensively reviewed elsewhere (Kelesidis et al., 2014). RANK-RANKL signaling has been shown to drive HIV replication in an NF-κB dependent fashion in acutely and chronically infected T cells (Kelesidis et al., 2014).

Fibroblast growth factor inducible factor (FN14) is a transient receptor to the TNF-like weak inducer of apoptosis (TWEAK/TWK) protein that is expressed on the cell surface in response to cellular injury (Winkles, 2008). The levels of soluble TWEAK in the plasma of treated and untreated HIV-infected individuals were found to be lower than in uninfected controls (Beltrán et al., 2014). FN14 expression in γδ T Cell subsets has been shown to be IL-21dependent (Vermijlen et al., 2007). Plasma IL-21 levels have been shown to be decreased in chronic HIV infection, an effect that is reversed in the presence of ART. Additionally, Elite controllers (patients exhibiting spontaneous immune control of HIV) have been shown to have higher circulating levels of IL-21. However, it has been seen that infected individuals may harbor higher levels of circulating IL-21 positive CD4+ T cells (Pallikkuth et al., 2012). While it is yet to be clearly established that FN14 expression is significantly dysregulated in HIV infection, the concurrent downregulation of TWEAK and cytokines such as IL-21 which may stimulate FN14 production suggest that FN14 signaling may represent a feasible therapeutic target for latency reversal.

CD30 levels in the plasma of HIV infected individuals have been found to be directly related to disease progression (Biswas et al., 1995; Biswas et al., 2006). It was recently demonstrated that in HIV infection, CD4+ T cells express higher levels of CD30 on their surface, regardless of ART status, whereas levels of soluble CD30 were only elevated in the viremic group. HIV RNA was seen to be enriched in the CD30 positive CD4+ T cell subsets from the blood of both untreated and treated patients, and also coincided with magnitude higher levels of HIV DNA. mRNA levels in the gut associated lymphoid tissue was also seen to co-localize with CD30 expression. The same study also utilized a clinically relevant anti-CD30 antibody, brentuximab vedotin, to treat ex-vivo PBMCs from HIV infected individuals which was seen to reduce the total amount of HIV-1 DNA (Hogan et al., 2018).

OX40 (CD134) ligation by its cognate ligand has been described to drive HIV replication in an NF-κB driven manner (Takahashi et al., 2001). In the setting of ART, OX40-expressing cells were observed to harbor significantly higher HIV DNA copy numbers and higher levels of clonally expanded HIV DNA. Intact proviruses were also seen to be enriched in the OX40-positive cells in four of the five subjects analyzed (Kuo et al., 2018).

### Activation of intracellular regulators of ncNF-κB during HIV infection

NF-κB inducing kinase (NIK), the primary protein involved in the ncNF-κB pathway, has been demonstrated to favor HIV replication. HIV Tat has been described to facilitate NIK mediated IKKβ activation, and it has been observed that knockdown of NIK leads to the inhibition of Tat driven HIV transcription (Zhou et al., 2008; Li et al., 2001).

Cellular inhibitor of apoptosis 2 (cIAP2) another key regulatory protein in the ncNF-κB cascade was identified to be able to directly inhibit HIV transcription through the inhibition of the ncNF-κB signaling pathway (Pache et al., 2015).

Signal transducer and activator of transcription 3 (STAT3) has been described to be activated and phosphorylated by different HIV proteins. HIV Gp120 has been described to drive STAT3-mediated NF-κB activation and cytokine production (Cicala et al., 2002; Del Cornò et al., 2014). HIV Nef has been described to activate and phosphorylate STAT3 in Macrophages, dendritic cells, and podocytes (Briggs et al., 2001; Husain et al., 2005). HIV Tat has been shown to cause STAT3 phosphorylation, dimerization, and nuclear translocation (Zeng et al., 2007; Jarboui et al., 2012).

Zinc finger protein 91 (Zfp91), which has been shown to associate with NIK and lead to p100 processing, was found to be involved in Tat mediated transcription, identified by siRNA mediated knockdown of Zfp91 leading to decreased Tat mediated HIV transcription (Faust et al., 2018).

Mucosa-associated lymphoid tissue lymphoma translocation protein 1 (MALT1), a Para-caspase that has been shown to promote TRAF3 ubiquitination has been demonstrated to favor a pro-replication profile, through the degradation of the MCPIP1 RNAse. It was demonstrated that MALT1 inhibition induced significant HIV-infected cell death and significantly impacted the level of HIV post reactivation with an LRA (Liu et al., 2013; Li H. et al., 2016).

Retinoic acid-inducible gene 1 (RIG1) is a positive regulator of the ncNF-κB pathway. In HIV infection, RIG1 activity has been demonstrated to be antagonized by HIV protease, with infection leading to decreased cytoplasmic RIG1 levels (Solis et al., 2011). On the contrary, it has also been described that HIV Vpr may acutely induce increased expression of RIG1 mRNA in human monocyte-derived macrophages (Zahoor et al., 2014).

TANK-binding kinase 1 (TBK1) is a negative regulator of the ncNF-κB cascade, and it has been demonstrated that HIV Vif and Vpr may bind to TBK1, preventing its autophosphorylation and the formation of a TRAF3-TBK1-IRF3 complex that is necessary for interferon production in human dendritic cells and macrophages (Harman et al., 2015). It is therefore plausible that HIV interactions with TBK1 may also influence TBK1-TRAF3 mediated NF-κB signaling, though this is yet to be established.

A20, an important negative regulator of NF-κB, is downregulated in intestinal epithelial cells during HIV infection due to the effects of interferon alpha, rendering cells more susceptible to cytokine mediated cell death. This downregulation was seen to be reversed following ART therapy in HIV infection (Chitre et al., 2018). It is possible that a similar upregulation could exist in cells that harbor the latent reservoir, allowing for transcriptional silence. Importantly, there has been evidence that the PKC agonist Prostratin upregulates A20 when used for latency reversal, leading directly to NF-κB inhibition, an effect that may be abrogated when co-administered with HMBA (Chen et al., 2016).

As evidenced above, HIV-induced modulation to the regulators of ncNF-κB signaling plays a significant role in developing strategies to target HIV for latency reversal, either through the potentiation of positive regulatory effects or the inhibition of the negative inhibitory effects.

## Shock and kill and the non-canonical pathway

Current latency reversal agents that are under investigation for “shock and kill” include, but are not limited to, Histone deacetylase inhibitors (HDACis), Bromodomain inhibitors, DNA methyltransferase inhibitors, proteosome inhibitors, Protein kinase C (PKC) agonists, WNT inhibitors and second mitochondria-derived activator of caspases (SMAC) mimetics. Nearly all of these agents have been shown to reactive latency *invitro* or *in vivo* with minimal effect on depleting total viral reservoir size (Kim et al., 2018). It has been shown that many of these agents have effects on the canonical NF-κB pathway. However, due to the generally proinflammatory nature of NF-κB and the significantly varied downstream effects of the canonical pathway, there is cause for concern with regards to off-target toxicity (Pache et al., 2015). In this scenario, selective agents targeting the ncNF-κB pathway would be preferred to mitigate toxicity risks.

SMAC mimetics are a group of drugs that have been shown to selectively target and inhibit cIAPs, allowing for downstream ncNF-κB signaling. The SMAC mimetics SBI-0637142 and LCL161 have been demonstrated to drive HIV reactivation through non-canonical signaling in cell lines. Additionally, in combination with HDAC inhibitors, they were able to reactivate latent HIV from ex *vivo* CD4+ T cells (Pache et al., 2015). Subsequently, a bivalent SMAC mimetic, Ciapavir, was shown to reactivate HIV in an *in vivo* mouse model, at the bone marrow level, in four out of the six mice treated with the agent, in the setting of suppressive ART (Pache et al., 2020). Debio 1143, another IAP antagonist, was shown to reactivate HIV from latency through the potentiation of the ncNF-κB pathway (Bobardt et al., 2019).

The SMAC mimetic AZD5582 which functions through the ncNF-κB pathway has been the focus of multiple recent studies and was recently demonstrated to reactivate HIV in a mouse model and in SIV infected macaques (Nixon et al., 2020). It is of note that minimal off-target effects were observed in these studies. The effect of AZD5582 was seen to be potentiated by crotonylation, allowing for superior latency reversal along with significant increases in p52 protein levels (Li et al., 2021). The effects of AZD5582 were also seen to be synergistically enhanced by selective and pan BET domain inhibition in a cell-line model of latency, however this was not seen to consistently result in HIV latency reversal in ex-vivo primary CD4+ T cells (Falcinelli et al., 2022). A recent study, examined the combination of AZD5582 with the DEAD-box polypeptide 3 (DDX3) inhibitor FH1321. It was observed that AZD5582 alone and in combination with DDX3 inhibition resulted in robust HIV reactivation in *in-vitro* Jurkat models and resulted in reservoir depletion in ex-vivo studies using PBMCs from HIV infected individuals (Jansen et al., 2023).

Interestingly, the latency reversal effects of AZD5582 differed between SIV-infected, ART-suppressed infant rhesus macaques and adult macaques, revealing lower levels of on-ART plasma viremia. In the same study, transcriptomic profiling in the infant macaques revealed that the expression of the ncNF-κB signaling genes RELB and NFKB2 were not significantly increased, contrary to prior observations in adults (Bricker et al., 2022).

Combination therapy with AZD5582 and a cocktail of 3 HIVxCD3 DART molecules (having human A32, 7B2, or PGT145 anti-HIV-1 envelope (Env) specificities) in a SHIV infected macaque model failed to result in an observable decrease in the viral reservoir, possibly as a result of poor latency reversal, with none of the infected animals demonstrating detectable viremia. It was suggested that lower pre-ART viral loads, and low pre-intervention reservoir sizes may have affected the potency of latency reversal in this study (Dashti et al., 2020). A more recent study examined AZD5582 with or without the IL-15 superagonist, N-803, in combination with SIV Env-specific Rhesus monoclonal antibodies (RhmAbs). N-803 is a potent LRA (discussed separately below), which was seen to enhance AZD5582 driven latency reversal. The combination of the RhmAbs with AZD5582 ± N-803, was observed to cause differential SIV-DNA depletion in CD4+ T cells based on anatomic location. However, significant decreases in “total body” SIV-DNA in CD4+ T cells (graphed as the sum of all SIV-DNA results from blood, lymph nodes, bone marrow and gastrointestinal tract) were observed following treatment with RhmAbs + AZD5582 ± N-803. Once again, it was observed that the magnitude of latency reversal was directly associated with pre-ART viral loads and the post-ART SIV-DNA CD4+ T cell reservoir, suggesting that viral reservoir size may be a crucial determinant of the efficacy of AZD5582 (Dashti et al., 2023).

PKC agonists such as bryostatin and prostratin are amongst the most investigated class of drugs for HIV latency reversal, both *in vivo* and *invitro*. Studies have indicated induction of cNF-κB and HIV latency reversal by PKC agonists (Bullen et al., 2014; Jiang and Dandekar, 2015; Kim et al., 2018; French et al., 2020). PKC agonism by PMA in cell lines was seen to result in the activation of the non-canonical NF-κB pathway via the recruitment of RelB to the APOBEC3B promoter, increasing the expression of APOBEC3B, a protein that is becoming increasingly recognized as a dynamic modulator of HIV replication (Gillick et al., 2013; Leonard et al., 2015; Bandarra et al., 2021). Prostratin was also seen to upregulate APOBEC3B in primary CD4+ T cells, but the involvement of RelB was not defined (Sung and Rice, 2006).

The IL-15 super agonists have been under study as a potential latency-reversal agent that has been shown to reactivate HIV and prime latently infected cells for clearance by immune effectors. Notably, IL-15 was seen to increase gene sets involved in TNF signaling via NF-κB and in STAT3 signaling in bulk CD4+ T cells (Jones et al., 2016; McBrien et al., 2020). Additionally, IL-15 was also seen to increase the expression of NF-κB2 in bulk CD4+ T cells (extended data (McBrien et al., 2020)). As mentioned previously, STAT3 can independently activate p100 processing and lead to ncNF-κB signaling. This mechanism remains to be described in the context of HIV latency reversal by these agents.

Proteasome inhibitors such as Bortezomib and Ixazomib are now under study as latency reversal agents via the activation of the NF-κB pathway (Miller et al., 2013; Natesampillai et al., 2018; Li et al., 2019; Timmons et al., 2020; Alto et al., 2021; Cummins et al., 2021), and they have recently been studied in the clinical setting (Cummins et al., 2021). The generation of p52 has been described as proteasome dependent with studies demonstrating that inhibition of the proteasome results in decreased p52 protein (Heusch et al., 1999). It has also been very well established that ncNF-κB inhibition is crucial to the efficacy of proteasome inhibitors in multiple myeloma (Chauhan et al., 2011; Dash et al., 2020). It is still to be elucidated whether the ncNF-κB pathway may play a similar role for HIV latency reversal by proteasome inhibitors.

CD40 targeted antibodies are increasingly becoming relevant in oncology. Agonists lead to observable T cell activation and anti-tumor potentiation induced by dendritic cells, and it has been well established that CD40-signaling can potentiate the ncNF-κB pathway and that a CD40-blockade inhibits dendritic-cell induced HIV latency reversal. Targeted antibodies to potentiate the effects of CD40 on ncNF-κB activity therefore represents a feasible strategy to achieve latency reversal, but the concomitant effects on the canonical pathway need to be considered (Hostager and Bishop, 2013; Kristoff et al., 2019; Vonderheide, 2020).

OX40 agonistic antibodies have now become increasingly relevant in cancer therapy, and OX40 agonism has been shown to boost HIV replication (Takahashi et al., 2001; Linch et al., 2015). As mentioned above, OX40, MALT1 and BCL-10 are all positive regulators of the non-canonical pathway. OX40 agonism has been seen to recruit and activate MALT-1 (Linch et al., 2015; Israël and Bornancin, 2018). Additionally, OX40-expressing CD4+ T cells were preferentially enriched for clonally expanded HIV, and some subjects harbored higher quantities of intact Proviral DNA (Kuo et al., 2018). As mentioned above, MALT-1 favors a pro-transcriptional profile. Interestingly, another protein, NEDD4-binding protein 1 (N4BP1), was seen to inhibit HIV replication, a function that was antagonized by MALT1. MALT1-mediated N4BP1 degradation was seen to facilitate the reactivation of latent HIV proviruses (Yamasoba et al., 2019). Targeted efforts to boost OX40 signaling or MALT1 potentiation, either in an OX40-dependent or independent manner may therefore represent feasible latency reversal agents not only through the non-canonical NF-κB pathway but also through other protein interactions.

RIG-1, a positive regulator of ncNF-κB, was targeted through the RIG-1 agonist acitretin which increased HIV replication in *in-vitro* and *ex vivo* models, and lead to preferential HIV infected cell apoptosis. However, a second study failed to reproduce similar results, suggesting further research is necessary to examine RIG-1 as a shock agent (Li P. et al., 2016; Garcia-Vidal et al., 2017). The interactions of these agents with the nc-NF-kB pathway has been summarized in **Table 2**.

**Table 2:** Shock and kill agents and their known effects on the non-canonical pathway.

Therapeutic agent	Effect on non-canonical signaling	Known drug effect in HIV	References
SMAC Mimetics	Inhibition of cIAP1	Latency reversal *in-vitro* and *in-vivo*	(Pache et al., 2015; Bobardt et al., 2019; Dashti et al., 2020; Nixon et al., 2020; Pache et al., 2020; Li et al., 2021; Bricker et al., 2022; Falcinelli et al., 2022; Dashti et al., 2023; Jansen et al., 2023)
PKC Agonists	Upregulation of A20 Recruitment of RelB to increase APOBEC3B	PKC agonist Prostratin upregulates A20, leading directly to NF-κB inhibition. PMA is a potent LRA	(Spina et al., 2013; Chen et al., 2016)
IL-15 super agonists	Increased expression of NF-κB Increase in TNF mediated NF-κB activation and STAT3 signaling	Latency reversal *in-vivo* following CD8 depletion and priming of latent cells for immune clearance	(Jones et al., 2016; McBrien et al., 2020)
Proteasome inhibitors	ncNF-κB signaling crucial for efficacy	Latency reversal and reservoir depletion *in-vitro* and *in vivo*	(Natesampillai et al., 2018; Li et al., 2019; Dash et al., 2020; Alto et al., 2021; Cummins et al., 2021)
OX40 agonist	Potentiation of MALT1-BCL10 action	Increases replication	(Takahashi et al., 2001; Linch et al., 2015; Kuo et al., 2018; Yamasoba et al., 2019)
RIG-1 agonists	ncNF-κB activation	Increases transcription *in-vitro* and *ex vivo.* (or) No effect	(Li P. et al., 2016; Garcia-Vidal et al., 2017)
MALT1 inhibition	Blockade of MALT-1 dependent MCPIP cleavage	Reduced levels of HIV post LRA treatment	(Liu et al., 2013; Li H. et al., 2016)

The above table aims to summarize known interactions of drugs being currently investigated for HIV cure, as part of “Shock and Kill”, and the NF-κB signaling pathway.

## Conclusion

Current ART therapy is incapable of achieving either a sterilizing or a functional cure of HIV. The current “shock and kill” strategies to eradicate HIV may involve the ncNF-κB pathway. As further studies in these fields progress, the role of this pathway may become better defined. Efforts to identify specific agonists of this pathway would significantly enhance “shock and kill” efforts and may ultimately contribute to the cure of HIV.

## Author contributions

AC: Conceptualization, Visualization, Writing – original draft, Writing – review & editing. MM: Writing – original draft, Writing – review & editing. AB: Funding acquisition, Resources, Supervision, Visualization, Writing – review & editing, Conceptualization.
